# A Role for Data Science in Precision Nutrition and Early Brain Development

**DOI:** 10.3389/fpsyt.2022.892259

**Published:** 2022-06-23

**Authors:** Sarah U. Morton, Brian J. Leyshon, Eleonora Tamilia, Rutvi Vyas, Michaela Sisitsky, Imran Ladha, John B. Lasekan, Matthew J. Kuchan, P. Ellen Grant, Yangming Ou

**Affiliations:** ^1^Division of Newborn Medicine, Boston Children’s Hospital, Boston, MA, United States; ^2^Fetal-Neonatal Neuroimaging and Developmental Science Center, Boston Children’s Hospital, Boston, MA, United States; ^3^Department of Pediatrics, Harvard Medical School, Boston, MA, United States; ^4^Abbott, Columbus, OH, United States; ^5^Department of Radiology, Boston Children’s Hospital, Boston, MA, United States

**Keywords:** brain development, data science, magnetic resonance image, neonate, nutrition

## Abstract

Multimodal brain magnetic resonance imaging (MRI) can provide biomarkers of early influences on neurodevelopment such as nutrition, environmental and genetic factors. As the exposure to early influences can be separated from neurodevelopmental outcomes by many months or years, MRI markers can serve as an important intermediate outcome in multivariate analyses of neurodevelopmental determinants. Key to the success of such work are recent advances in data science as well as the growth of relevant data resources. Multimodal MRI assessment of neurodevelopment can be supplemented with other biomarkers of neurodevelopment such as electroencephalograms, magnetoencephalogram, and non-imaging biomarkers. This review focuses on how maternal nutrition impacts infant brain development, with three purposes: (1) to summarize the current knowledge about how nutrition in stages of pregnancy and breastfeeding impact infant brain development; (2) to discuss multimodal MRI and other measures of early neurodevelopment; and (3) to discuss potential opportunities for data science and artificial intelligence to advance precision nutrition. We hope this review can facilitate the collaborative march toward precision nutrition during pregnancy and the first year of life.

## Introduction

Optimal nutrition early in life is essential to support the rapid growth and development during infancy, especially among infants born preterm or with severe medical conditions. A critical period, from the 3rd trimester of pregnancy through the first 1,000 days of life, encompasses multiple simultaneous neurodevelopment processes that lay the foundation for increasing neural complexity and subsequent refinement (1, 2). The extended developmental timeline of the human brain provides a large risk surface for environmental factors to affect the developmental trajectory, either by increasing plasticity or vulnerability. While plasticity can provide an increased ability to repair injury, resilience factors can be superseded by damaging factors (3). Impaired brain development during this critical period can result in lifelong functional deficits and an increased risk of neurological disorders (4).

Nutrition plays a key role in supporting proper neurodevelopment. Adequate supply of both macronutrients and micronutrients is imperative to support the complex processes underlying brain development. Myelination accelerates during infancy (5) and is affected by intake of nutrients such as long-chain fatty acids, choline, iron, sphingomyelins and folic acid (6). At 2 years of age, synaptic density peaks are 50% higher than in adulthood (7). Tremendous energy in the form of macronutrients is required to fuel such extensive development. In fact, brain glucose consumption at 2 years of age is already equivalent to that of the adult and continues to rise during childhood, accounting for 50% of the basal metabolic rate by age 10 (8). Malnutrition during the prenatal and postnatal periods inflicts a tremendous cost on the welfare and lifetime achievement of the individual, and therefore to society as well. Indeed, if the three most common micronutrient deficiencies (zinc, iron and vitamin A) were corrected, it is estimated that global IQ would increase by 10 points (9).

Advances in multimodal brain magnetic resonance imaging (MRI) is enabling precision nutrition – optimizing nutrition for individuals – which has the potential to provide high-impact nutritional interventions (10). Multimodal MRI provides insights on the nutritional requirements of specific developmental stages, and progress to the ability to provide individual recommendations. Several nutrients have been specifically studied for early life impacts on neurodevelopment (Table 1) when supplemented after birth. Currently, we have limited knowledge regarding how nutrients interact with other nutrients and non-nutritional factors to modify health outcomes. Understanding these interactions will improve recommendations for populations and individuals. This paper surveys mother-infant nutritional studies, focusing on emerging data science opportunities toward precision nutrition in term infants. Part I reviews current knowledge on the effects of factors (nutritional, genetic and environmental) on infant brain development. Part II discusses data science opportunities in multimodal MRI and marker studies for nutrition. Part III lists major databases, trials and resources relevant to data-driven precision nutrition.

**TABLE 1:** Selected nutrients studied for neurodevelopmental effects during infancy.

Nutrients	Age of exposure	Age of effects	Participants	Outcome measures	Preterm/term	Country	References
Choline	Birth	7 years	895	Visual memory	P/T	United States	(206)
	12 weeks gestation–3rd trimester	7 years	26	Color-location memory task	T	United States	(191)
	3rd Trimester	13 months	24	Processing speed, visuospatial memory	T	United States	(192)
	2nd trimester–3 months postpartum	40 months	49	Attention and social withdrawl	T	United States	(193)
DHA	18 weeks gestation–3 months postpartum	4 years	76	Mental processing	T	Norway	(194)
	3-4 months postpartum	6 months	55	Recognition memory	T	United States	(208)
	28 weeks gestation	12 months	126	Problem solving/IQ	T	Norway	(195)
	18 weeks gestation to Birth	5 years	797	Attention	P/T	Mexico	(105)
Folate	30 weeks gestation	10 years	536	Cognition	T	India	(214)
	20 weeks gestation–Birth	8.5 years	59	Executive function	T	Germany, Spain, Hungary	(56)
	Gestation	1 month	1186	Development quotient	T	China	(196)
	Birth–3 months	6 months – 5 years	150	Myelination, behavioral development	T	United States	(210)
Iodine	1st Trimester	3 – 18 months	194	Psychomotor development	T	Spain	(58)
	Preconcep-tion	6 – 7 years	654	Intelligence	T	United Kingdom	(39)
Iron	Gestation	6 months	965	Mental development index	T	South Korea	(197)
	34 weeks gestation	18 months	331	Motor development	T	Spain	(198)
Lutein	3-4 Months postpartum	6 months	55	Recognition memory	T	United States	(208)
	1st-2nd Trimester	7 years	1093	Verbal	P/T	United States	(211)
Vitamin B12	12 months	12 months	183	Motor development	T	Guatemala	(199)
	Preonception	2 years	74	Cognition and language	T	India	(209)
	1 – 21 months	1 – 21 months	112	Early neurological development	T	Turkey	(200)
Vitamin D	13 weeks gestation	14 months	1820	Psychomotor development	T	Spain	(201)
	32 weeks gestation	6 months	960	Language development	P/T	Vietnam	(202)
	2nd Trimester	24 months	1020	Receptive language development	P/T	United States	(203)
Zinc	6 months	6-18 months	251	Habituation, attention	T	Peru	(204)
	Birth-3 months	Term corrected	100	Attention	P	India	(205)

## Maternal Factors Influencing Infant Brain Development

**FIGURE 1:**
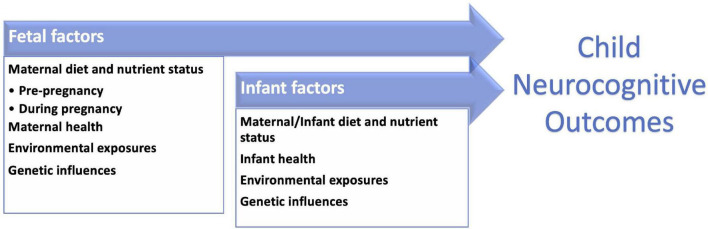
Neurodevelopmental outcomes in childhood are influenced by multiple factors including those present in the prenatal period and infant stages.

Maternal diet plays a crucial role in nutritional status before and during pregnancy, and in breastmilk nutrition during lactation (1, 11–19). These factors, along with parental, environmental, genetic, socioeconomic, and other variables, influence infant brain development and neurocognitive functions later in life (20–22; Figure 1). The process is multifactorial and complex. For decades, multidisciplinary research has aimed to identify key dietary influences during pre-conception, pregnancy, and lactation that can be the basis for efforts to improve infant health and development (Table 1; 23–26). Likewise, researchers have studied developmental differences between breastfed and formula-fed infants driven by extremely complex nutritional differences between breast milk and infant formula and also inherent non-nutritional differences between those populations. There are many societal factors that can facilitate or hinder breastfeeding, so differences between breastfeeding and formula feeding could also result from these non-nutritional influences. While those studies have been successful in determining the essential role of many nutrients such as folate and iodine that have significant consequences when in deficit, new analytical approaches open the door to understanding the complex interactions between dietary nutrients and designing individualized dietary recommendations that account for subtle effects and complex interactions between dietary nutrient and non-nutritional factors. Further, existing studies on early nutritional influences mostly focus on preterm infants (23, 27–33), due to the high risk of nutritional deficiencies and neurodevelopmental concerns in this vulnerable population. Term-born infants, on the other hand, face different nutritional needs (34, 35), and warrant specific studies.

### Pre-conception

The maternal diet prior to conception affects pregnancy outcomes and infant neurodevelopment (14, 16, 17, 36). Prenatal deficiency in micronutrients such as folate, iron, and iodine have been shown to impact infant outcomes. For example, maternal pre-conception folate supplementation was found to reduce neural tube defects (17). Offspring of mothers that received a pre-conception supplement of folic acid and iron displayed improved fine motor scores at 24 months of age compared to children of mothers that received folic acid alone (37). Iodine deficiency can negatively impact the development of the cerebral cortex and axonal connections, as well as the myelination of the central nervous system (38). Low pre-conception maternal intake of iodine is associated with lower IQ in school-aged children aged 6-7 years, emphasizing the long-lasting impact of pre-conception nutrition deficiencies (39). In addition to specific nutrients, the activity of metabolic pathways can be measured pre-conception. Higher maternal homocysteine levels, reflective of sub-optimal one-carbon metabolism, were associated with an elevated risk of anxiety, depression, and social problems at school age (40). However, our current knowledge is very general and does not incorporate individual patient nutritional needs. Future work to understand interactions between micronutrients, as well as differences in individual responsiveness to nutrient deficiency or excess, is needed to effectively assess the value of and potential targets for nutritional intervention in the pre-conceptual period.

### During Pregnancy

During pregnancy, maternal intake of nutrients such as folate, iron, iodine, zinc, choline and polyunsaturated fatty acids can promote the development of the brain and central nervous system in infants (41). Folic acid supplementation is associated with increased brain volume as well as increased performance in language, cognitive (42) and visuospatial domains (43). However, questions remain about how the accumulation of unmetabolized folic acid from high intake may impact fetal development (44). Intrapartum maternal iron deficiency leads to decreased fetal iron stores (45), which can persist for many months after birth (46). Infants with iron deficiency at birth have lower language and fine motor scores at 5 years of age (47). Iron deficiency during infancy leads to impaired inhibitory control in later childhood (48) and poorer cognition and socio-emotional function through adolescence and young adulthood (49, 50). Iodine deficiency is estimated to affect as many as 2 billion people worldwide and is the leading cause of preventable impaired mental functioning (51). Fetal iodine deficiency is associated with irreversible visual and cognitive impairment, as well as worse gross motor development (52). Gestational zinc deficiency has been associated with decreased novelty preference (53), though clinical trials have failed to demonstrate positive effects of maternal zinc supplementation upon child neurodevelopment (54). Choline is essential for neurodevelopment, and maternal choline intake is positively associated with child neurodevelopmental outcomes, including processing speed, attention, inhibitory control, and spatial memory (55). Inadequate gestational supply of nutrients such as folate and polyunsaturated fatty acids (docosahexaenoic & linolenic acid) can impair brain development (56). Given the growing evidence base for the role of maternal intrapartum nutrition on infant brain development, it is an ideal time to apply precision medicine approaches in future studies.

### Lactation

During early lactation, an infant is dependent solely on the nutrients from maternal breast milk for nutrition and calories (21). Proper nutrition remains critical in the first years of life to support brain development, including intake of iodine, choline, polyunsaturated fatty acids and other nutrients (1, 3). Key nutrients such as iodine continue to be important during lactation. Maternal supplementation of iodine in populations with high rates of deficiency has been shown to lower infant mortality rates improve cognitive function (57). Additionally, maternal supplementation with iodine during lactation improved psychomotor development (58). A recent systematic review concluded that preclinical and clinical evidence indicates choline supplementation of maternal and/or child diets during the first 1,000 days of life supports normal brain development and increases resilience to developmental insults (59). Other studies are beginning to clarify the role of omega-3 fatty acids in early development. A study of docosahexaenoic acid (DHA) supplementation during infancy demonstrated higher gesture scores at 12 and 18 months (60). Recently, multivariate analysis identified positive associations between maternal intake of omega-3 fatty acids and sub-regional volumes in the frontal cortex and corpus callosum at 1 month of age among a cohort of 92 term-born breastfeeding mother-infant dyads (61). One ongoing application of precision nutrition during lactation is the targeted supplementation of fat and protein using milk analysis for hospitalized infants (62). With advances in the availability of milk analysis, individualized supplementation of expressed human milk could improve growth and developmental outcomes among high-risk infants.

### Breastfeeding

A common question is how the relative exposure to breastmilk and formula influences infant neurodevelopment. Breastfeeding exposure has consistently correlated with neurodevelopmental differences in school-aged children. Neurocognitive studies found significantly lower IQ scores as measured by Kaufman Brief Intelligence Test and Wechsler Abbreviated Scale of Intelligence among school-aged children who were fed formula as infants (63–65). These studies, together with others, quantified a neurodevelopmental benefit to breastfeeding during infancy but did not identify any specific nutrients responsible for the association.

Multimodal brain MRI correlates of these neurodevelopmental outcomes are a first step toward defining potential biomarkers. Diffusion MRI studies found lower fractional anisotropy values (i.e., more diffuse water flow) in left hemisphere white matter regions among 18 formula-fed male children compared to 10 breastfed male children at 8 years of age (66). As fractional anisotropy is sensitive to white matter integrity, and could be an early biomarker of the impacts of breastfeeding on brain development. Emphasizing the role of non-nutritional influences, there was no observed effect among female children. Structural MRI studies at the same age also demonstrated lower gray matter volume in the left inferior temporal and superior parietal lobes of 10 male infants who were fed formula compared to 10 male infants who were breastfed (67). Two larger studies extended these findings, identifying lower volumes across the whole brain, total gray matter, cortical gray matter, and subcortical gray matter among ∼10-year-old children who were fed formula during infancy (N = 148) (65); and thinner cortical surfaces in the superior and inferior parietal lobes at 12-18 years of age among 301 children who were fed formula as infants compared to 270 breastfed infants (64). Besides, specialized brain MRI approaches to quantify myelination (68) identified earlier and more rapid myelination in the frontal and temporal white matter, peripheral aspects of the internal capsule and corticospinal tracts, superior longitudinal fasciculus and superior occipital-frontal fasciculus from 10 months to 4 years of age in breastfed infants (69). These studies highlight potential neuroanatomical biomarkers for previously observed neurocognitive associations with breastfeeding (70). However, currently identified markers often have weak effect size and limited ability to predict outcomes. But group-level information about brain structure can be an unparalleled tool to understand the dynamic, temporal nature of nutrient effects on brain development and confounding factors.

While the above studies were cross-sectional, longitudinal data can determine how nutritional exposure alters the trajectory of neurodevelopment. One study of term infants included brain MRI measures at 6, 12, 18, 24, 36, 48, and 60 months, totaling 231 MRI scans from 39 breastfed infants and 221 MRI scans from 54 formula-fed infants (6). Improved overall myelination in the white matter and corpus callosum was found in breastfed children, and the difference increased in magnitude from birth to about 2 years of age then plateaued thereafter. Neurocognitive scores as measured by Wechsler Abbreviated Scale of Intelligence were significantly higher in general, verbal, and non-verbal cognitive domains among breastfed infants. Also, when the formula-fed cohort was divided by formula composition, the formula-based intake of sphingomyelin, phosphatidylcholine, folic acid, iron, choline, vitamin B12, and two omega-3 fatty acids (DHA and arachidonic acid) were associated with myelination differences in focal brain areas. Future studies need to address how breastfeeding alters development during 0-6 months of age, when the brain develops most rapidly.

### Non-nutritional Factors

Environmental influences on human neurodevelopment, both positive and negative, begin prenatally (12, 22, 71, 72). Preconception, non- nutritional influence from both parents can shape infant development (73). Maternal adverse psychosocial exposures before conception can influence child development (13, 74–76). Potential mechanisms for the transfer of maternal stress responses to the fetus include transplacental passage of molecular factors, alterations to the uterine environment, and epigenetic changes (73, 77, 78). Postnatally, adverse experiences during childhood are associated with altered brain architecture as well as adverse behavioral and educational outcomes (79–81). For example, childhood poverty is associated with decreased executive function skills; further, childhood cortisol levels were correlated with poverty and measures of executive function (80). Other environmental exposures including toxins, can negatively affect neurodevelopment (82): exposures to endocrine-disrupting chemicals can increase the risk of adverse outcomes such as neurobehavioral disorders (78). Finally, nutritional influences on neurodevelopment are modified by exposures to inflammation, both related to adverse experiences as well as systemic illness or changes in the microbiome (15, 83, 84). For example, iron is sequestered as part of the inflammatory response, with chronic inflammation resulting in iron deficiency anemia in vulnerable populations (85, 86).

Maternal health also influences long-term child neurodevelopment. There are critical windows such as the first trimester, when maternal endocrinological conditions, such as abnormal thyroid hormone levels, can affect brain development (87). A cohort of school-aged children born to women with untreated hypothyroidism had lower neuropsychiatric testing scores across several domains including language and visual-motor performance, compared to peers without maternal hypothyroidism (88). Further, maternal conditions such as obesity and pre-eclampsia alter the placental transfer and fetal accumulation of omega-3 fatty acids (89). Specific dietary factors may be most beneficial among infants affected by maternal conditions.

Genetic predispositions can also affect infant neurodevelopmental outcomes, and modifies the effect of other exposures (90). For example, the presence of a maternal polymorphism in peroxisome proliferator-activated receptor gamma (*PPARG*), which leads to decreased protein activity, was associated with lower scores in cognitive, language, and motor realms in offspring at 18 months of age (91). The child’s *PPARG* allele status was not correlated with these outcomes, indicating the lasting influence of the intrauterine environment on long-term development in childhood. Exposure to toxins, *in utero* and/or postnatally, can negatively impact neuropsychiatric development. For example, in a Taiwanese cohort of 181 children, higher cord blood mercury concentrations were associated with lower cognitive scores at 2 years of age, most significantly among children with a high-risk allele of Apolipoprotein E (92). Similarly, the relationship between child motor development at 20 months of age and maternal blood mercury concentrations were affected by common variants in glutathione-related genes (93). Common variants in glutathione metabolism genes also increased the negative effect of intrapartum maternal exposure to secondhand smoke on cognitive performance, as measured by the Bayley Scales of Infant Development at 2 years of age (94). With such an array of potentially influential factors on child development, a nuanced approach that customizes positive factors such as nutrition may be most effective if personalized based on co-existing risk factors.

## Data Science Opportunities Toward Precision Nutrition

The rise of data science, including the application of artificial intelligence to big data, offers unique opportunities for nutritional science. We can generate new hypotheses that can drive intervention studies testing combinations of nutrients and other factors. In addition, machine learning can also enable the analysis and interpretation of large-scale clinical studies. While much interventional nutrition research focuses on one or a few nutrients (95), modern data science allows us to consider multiple nutrients together and quantify their complementary or competing contributions to outcomes. Nutritional intervention studies typically involve only one or a few nutrients, while retrospective correlation studies can examine multiple nutrients, these nutrients are often analyzed separately. Further, only a limited set of non-dietary factors (96, 97) (environmental, genetic, socioeconomic, etc.) have been typically included in models of nutritional effects. In contrast, machine learning approaches can jointly consider all these factors to identify biomarkers of important outcomes, or to determine influential dietary and/or nutritional supplements given other features of an individual mother-infant dyad such as genetic risk, environmental exposures, or previous nutritional interventions. Big data in this context could refer to large cohorts, large number of variables per participant, or both. Such approaches fit well with the concept of *precision nutrition* (98) – nutrition that can be optimized for individuals, which is elaborated in the 2020-2030 Strategic Plan for National Institute of Health (NIH) Nutrition Research as released in May 2020 (99).

A challenge of the traditional approach to nutrition studies of neurodevelopment is that, in addition to measuring the efficacy of a particular nutrient dose, the temporal window for optimal influence must also be assessed for each nutrient or combination of nutrients. In many cases, a nutritional deficiency may exist, even in healthy and term-born infants, but the effects of the deficiency may not be evident given the long time between exposure and measurable outcome, requiring highly sensitive and specific biomarkers to be identified (100–102). Traditionally, the impact of infant intake of a single nutrient on neurodevelopmental outcomes, or a biomarker of that outcome, is followed over time, typically in school age, childhood, or adolescence (28, 50, 63, 67, 103). This long interval between exposure and assessment is necessary because many neurodevelopmental outcomes cannot be measured until school age or adolescence. For example, the varying impact of supplementation with DHA on neurodevelopment (104–107) may be clarified using machine learning to improve sensitivity by accounting for confounding factors. The need for early intervention fuels studies of blood biomarkers or non-invasive imaging. Often, nutrition studies require highly sensitive and specific biomarkers (100–102) because the effects of suboptimal nutritional status are only detectable after a long post-exposure latency. Such markers could enable the optimization of nutrition at the earliest time possible for mothers and infants. Finally, combining longitudinal data using machine learning could develop new hypotheses on nutrient combinations that impact markers or outcomes.

### Multimodal Brain MRI

Neuroimaging such as multimodal brain MRI and magnetoencephalography can non-invasively and quantitatively measure localized effects of specific nutrient on brain structure and function (26, 32, 61), identify neuroanatomic mechanisms linking nutritional status with brain development outcomes, and evaluate the effects of nutritional interventions (29, 100–102). Neuroimaging provide objective and quantitative outcomes that compliment other nutrition biomarkers such as reported diet. Disadvantages include logistical hurdles and expense of image acquisition, and the requirement of advanced analytics. At least two recent advancements are facilitating the use of brain MRI in nutritional studies. The first is the rise of brain MRI databases that span the human lifespan (100–102). Hundreds to tens of thousands of brain MRI data from healthy controls are now available publicly, many with nutrition, parental, socioeconomic, behavior, neurocognitive development, environment, and other types of comprehensive data. The other advancement, as elaborated later, is the quantification of normal brain development in space across different anatomic locations, in time across ages, and across modalities that alternatively assess structural, diffusion, metabolic, or functional data (108). These increase our ability to pinpoint the subtle and complex patterns associated with specific nutrients or malnutrition.

Multi-modal brain MRI offers comprehensive tools to evaluate the effect of nutrition on the brain structure and function. Structural, or anatomic, brain MRIs such as T1- and T2-weighted sequences help reveal nutritional effects on regional volumes (61, 109–112) as well as cortical surface thickness, area, curvature and folding patterns (113–115). T2* and T2’ sequences, in particular, have shown potential to directly quantify iron levels in the brain (116, 117), as has quantitative susceptibility mapping (117–119). Diffusion-weighted and diffusion-tensor brain MRIs offer a lens to quantify white matter water diffusion. Diffusion-tensor-based parameterization maps such as fractional anisotropy, mean diffusivity, apparent diffusion coefficient, radial diffusivity, and axial diffusivity images measure water diffusion magnitude and directionality. Diffusion-tensor-reconstructed tractography quantifies the structural connectivity along white matter tracts (32, 109, 120, 121). Functional MRI of the brain, on the other hand, measures the brain circuits/connectivity based on blood flows, and have been used to probe the neural substrate of nutrition on neurodevelopmental outcomes (122–125). Magnetic resonance spectroscopy reveals the metabolism in the brain that can be related to nutrition (126–129). The choice and the optimal combination of various brain MRI sequence remain an open topic, though, and are largely dependent on the cohort, specific nutrients, and study aims (100, 102).

### Neurophysiology Tools

Brain activity and its developmental trajectory can be monitored non-invasively using scalp electroencephalography (EEG) or magnetoencephalography (MEG). EEG/MEG recordings are non-invasive procedures that can be performed in subjects of any age or clinical status. This makes them suitable tools for testing the short-term and long-term neurophysiological effects of malnutrition or nutritional interventions.

EEG is sensitive to the neural correlates of cognitive dysfunction associated with early childhood malnutrition (130). Childhood malnutrition, before the age of two years, is shown to lead to irreversible shifts in the cerebral activity organization that are measurable through EEG (131–134). Quantitative EEG biomarkers of the malnutrition effects may help stratify children at risk of adverse neurodevelopmental effects and inform targeted interventions. An EEG-based age-adjusted classifier (mainly driven by the EEG alpha activity in the lingual gyrus) was also recently developed, which distinguished infants with histories of protein-energy malnutrition from healthy controls with 82% accuracy (134). This encourages the use of EEG as a mediator in disease progression models to optimize cost-effective interventions.

A demonstrated association was also shown between infant diet and early postnatal development of gamma EEG activity, which is sensitive to the development of the GABAergic system (135). EEG activity during infancy was also shown to differ between infants who are fed through breastfeeding versus milk or soy formula, possibly suggesting that EEG measures may reflect environmental- or diet-related influences on the development of brain function that could lead to different neurodevelopmental trajectories (136). Nutritional exposures outside of infancy also lead to differences in EEG traits. Resting-state EEG activity is altered by the content of omega-3 and omega-6 fatty acids diets in infants, children, and adults (100).

With a coordinated effort, large EEG datasets could become available as EEG technology is widely used in hospital settings and can be recorded at the bedside. The use of high-resolution EEG techniques or even MEG, as well as the use of recent advanced computational methods for data analysis, can potentially enhance the development of neurophysiological markers of nutritional interventions or disorders. To our best knowledge, no MEG studies exist investigating these aspects. Nevertheless, the increasing availability of MEG systems worldwide will likely bring to wider, multi-center collection of MEG data in this direction.

### Non-imaging Biomarkers for Early Nutritional Interventions

As the time window for nutritional interventions to optimize infant neurodevelopment is likely to precede measurable outcomes, proximal biomarkers of neonatal nutritional status are necessary. Potential markers of infant nutritional status include weight, head circumference, body composition, and macular pigment density (137–145). Body composition measurements determine the relative amounts of fat and fat-free mass, the latter of which includes total body water, bone mineral, and muscle mass (146). Body composition can be measured by multiple approaches including anthropometric measurements, isotope dilution, dual energy x-ray absorptiometry, bioelectrical impedance analysis, MRI, and air displacement plethysmography (147). Each measure has varying measurement types, degrees of accuracy, measurement time, expense, and associated exposures. Differences in body composition among preterm infants have been associated with speed of processing at preschool age (148), indicating that body composition has promise as a proximal biomarker of nutrient effects on neurodevelopment. In addition, regional bone mass (mineral and density) such the head region measured with dual energy X-ray absorptiometry (DEXA) which correlates very well with head circumference measures may potentially serve as a non-invasive marker for brain development.

Macular pigment optical density (MPOD) often reflects lutein and zeaxanthin intake in the adult population (137, 149, 150). It is quantitative and non-invasive measure that can be followed serially over time. Studies have demonstrated the feasibility of measurement among preterm infants (151) and a positive correlation between maternal serum zeaxanthin levels and infant MPOD measurements (152). Breastfed infants have higher MPOD values than formula-fed infants (153, 154), though the exact milk components responsible for this difference remain to be determined. Childhood MPOD measures have been associated with academic performance, supporting its potential role as a biomarker of neurodevelopment (155). However, open questions include (a) whether MPOD remains stable during lactation, and the lack of longitudinal studies in a large cohort for the dynamics of MPOD from pre-conception, pregnancy, and postpartum; (b) what exact nutrients or nutrient combinations in these three stages do MPOD reflect in maternal nutrition – because existing evidence that MPOD reflects lutein and zeaxanthin intake has been from non-pregnant populations. This association may or may not replicate in mothers before, during, and after pregnancy as dietary carotenoids are also transferred to breastmilk (153, 155) and therefore are likely to have a different relationship with MPOD (145).

### Measuring Nutritional Intake

Ascertainment of the effects of maternal nutrient intake on infant outcomes can be achieved through targeted supplementation of single or multiple nutrients, or by passive assessment of nutrient intake. Estimating nutrient intake from consumption of infant formulas can include significant error unless bottles are weighed before and after feedings. A formalized, quantitative method to profile maternal nutrient intake is food frequency questionnaire (FFQ) (156, 157). FFQs collect the portion size and frequency of consumption among a list of foods, and can be self-administered or completed by interview (158). This food data is then converted into daily nutrient intake values (159, 160). Advantages to FFQs include scalability, ease of administration, and opportunity for serial measurement. Disadvantages include concerns about reproducibility and systematic errors and biases associated with self-reported data, memory-based measurements, inability to verify or falsify data (161), or objective infant brain measurement (61), and data processing assumptions.

### Multivariate Analysis of Macro/MicroNutrient Interactions

The majority of existing studies focus on the effects of a single nutrient, or multiple nutrients examined independently. However, nutrients interact with each other to produce biological effects. For example, choline and DHA interact during eye and brain development (162); vitamin B12, iron and folate all affect red blood cell development (163); and lutein, vitamin E, arachidonic acid and DHA interact during brain development (164–166). Defining additional subsets of nutrients that jointly impact brain development and general health is an essential step toward precision nutrition (167). Multivariate analysis in machine learning and data science is appropriate for the task. One obvious challenge, though, is the need for big data including a full spectrum of nutrient, biomarker and outcome data for ideally thousands or more individuals.

### Pathway Connecting Maternal Diet, Breastmilk, Infant Brain Development and Later Neurocognition

Current mother-infant dyad studies focus on associations between nutrient intake and infant outcomes, but causality is not addressed by linking maternal dietary factors to changes in breastmilk composition. For instance, breastmilk components that influence infant brain health may be constant and not significantly modified by maternal intake of nutrients. Alternatively, the maternal nutrients associated with infant brain health may not be related to the quantity of breastmilk components but could instead vary based on the bioavailability or activity of nutrients. Furthermore, nutrient bioavailability and activity may vary during pre-conception, pregnancy, and lactation periods. One way to move toward a causality study is to find a pathway linking maternal diet, breastmilk components, and infant development (either neurocognitive measurements or biomarkers such as brain MRI measures, or both). Establishing a pathway can generate a specific hypothesis regarding nutrients or supplements that may impact breastmilk components and subsequently improve infant brain health. These nutrients could then be studied in randomized trials, the modification of which may show the maximum impact in infant brain development. Recent advancements in multivariate analysis, artificial neural network analysis, and graph theory set the stage for such a pathway study.

### Moving From Population Science to Precision Nutrition

Precision nutrition can produce tailored nutritional intake recommendations to optimize health and outcomes (168). By contrast, most nutritional studies focus on population-level associations. Whereas individuals differ by dietary habits, genetics, environmental exposures, parental factors, health status, microbiome, metabolism, socioeconomics, physical activity, psychosocial characteristics, and other factors, precision nutrition aims to create individual nutrition recommendations that consider these complex influences (169–171). Precision nutrition is an important constituent of precision medicine (98). The 2020-2030 NIH Strategic Plan for Nutrition Research articulated four strategic goals: (1) discover what we eat and how it affects us; (2) investigate what and when we should eat; (3) define how nutrition promotes health across our lifespan; and (4) understand how to modify diets to improve health, or “Food as Medicine” (99). The prenatal period, as reviewed in this paper, marks the earliest time that precision nutrition can make an impact, and arguably the time precision nutrition can make the largest lifelong impact.

## Existing Resources to Power Precision Nutrition

### Birth Cohorts

Some large birth cohort studies and national registries have been collecting data since the 1990s or earlier. These databases aim to provide information that advances our understanding of how various factors act together to impact psychical, socio-emotional, cognitive and behavior development of participants across infancy, adolescents, adulthood, and subsequent generations (172, 173). A typical birth cohort contains thousands to tens of thousands of participants, who were followed up for years or decades for these comprehensive sets of data (Table 2). In all these birth cohort studies, nutritional data is collected alongside other measures that are potential biomarkers of development. Some studies include brain MRI measurements, sometimes longitudinally collected. Examples of such MRI-containing birth cohorts are Dutch Generation R, FinnBrain, Norwegian MoBa, Singapore GUSTO cohorts. Merging data across cohorts, a big data opportunity not yet explored, may face problems such as missing or differently-formatted data. A subset of the datasets may share sufficient data characteristics to be tangible for meta-analysis.

**TABLE 2:** Birth cohort studies.

Cohort	Families	Year Started (Ended)
Avon Longitudinal Study of Parents and Children (ALSPC)	14,000	1990
Dutch Generation R	4,000	2001
United Kingdom Millennium Cohort Study (MCS)	21,000	2000
Norwegian Mother, Father and Child Cohort Study (MoBA)	100,000	1999 (2009)
Danish National Birth Cohort (DNBC)	100,000	1996-
Greek Mother-Child Study (RHEA)	1,600	2007 (2015)
Amsterdam-Born Children Development (ABCD)	12,000	2003
French EDEN Mother-Infant Study	1,800	2003
Growing Up in Australia	10,000	2002
Growing Up in New Zealand	7,000	2009
Growing Up in Scotland	10,000	2004
Growing Up in Ireland	18,000	2008
China Anhui Birth Cohort	17,000	2008
KUNO-Kids Germany	5,000	2019
China Shengjing Cohort	1,000	2019
Singapore GUSTO Birth Cohort	1,100	2009
Spanish INfncia y Medio Ambiente	3,600	1997 (2008)
United Kingdom Born in Bradford	30,000	2007
FinnBrain Research	3,000	2011 (2015)

### Quantification of Typical Brain Development Over the Lifespan

Healthy brain MRIs in the public domains have grown from ∼8000 in 2014 (174) to now more than 40,000, and including many that represent timepoints now across human lifespan (174–176). Ongoing projects include the HEALthy Brain and Child Development (HBCD) initiative, which is collecting comprehensive birth cohort data from thousands of mother-infant dyads in the next decade in more than 20 sites across the United States (177). Datasets that depict typical neonatal brain development through early childhood are increasingly available at a 1 mm x 1 mm x 1 mm spatial resolution and high temporal resolution (bi-weekly, monthly, quarterly and annual intervals) (178–181). Fetal brain atlases are now available at a weekly interval between 19 and 39 weeks of age (182). These valuable brain MRI data during typical development provide baseline measures for comparison in future cohorts, though the applicability of each reference dataset should have careful consideration of potential confounding exposures (183, 184). Computer-constructed atlas characterizes structural, diffusion and functional neurodevelopment at high spatial and temporal granularity (185). Machine learning-driven age prediction captures subtle deviation compared to a subject’s chronological age, reflecting accelerated or slowed changes associated with aging (186–188). These datasets and sensitive algorithms equip nutrition researchers with a better understanding of brain development in early life, and a series of tools to explore the relationship between maternal nutrition and infant brain health. Opportunities also emerge for brain MRI to identify subtle nutrition deficiencies and suggest nutritional supplements for individual mother-infant dyads, as well as to assess the influence of additional factors relevant to precision nutrition on sensitive MRI biomarkers of development.

### Ongoing Clinical Trials

While there are many clinical nutritional intervention studies, few collect the comprehensive data necessary for precision nutrition. At clinicaltrials.gov (accessed on 01/09/2022), there are 202 clinical studies currently enrolling participants that are identified by the keyword ‘nutrition’ and participant age of 0 years, 103 of which are interventional. There were an additional 82 active clinical studies, 49 of which are interventional. Ten active interventional trials involved dietary supplements, mostly focused on preterm infants (7 of 10). An additional 792 completed clinical trials were identified using the same search terms, of which 549 were interventional. One hundred thirty-two were focused on infants, but again only those born preterm, while only 2 included neurodevelopmental outcomes or utilized brain magnetic resonance imaging. Thus, research motivation for studying nutritional interventions for infants is high, but few studies focus on term infants, neurodevelopment or MRI analysis of brain structure or connectivity.

### National Institutes of Health Nutritional Task Force

The NIH Nutrition Research Task Force, formed in October 2016 (189), released a 10-year (2020-2030) strategic plan to accelerate nutrition research into precision nutrition in May 2020 (99, 190). To achieve the third of four NIH-wide strategic goals – “to define the role of nutrition across the lifespan” – three specific objectives were developed to focus on nutrition in the pre-conception, pregnancy and infancy stages. These objectives are: examine the role of peri-conceptional and prenatal nutrition in development and disease outcomes (Objective 3-1); enhance knowledge of human milk composition and the translational roles of its components (Objective 3-2); and access the influence of diet and nutritional status on infant developmental and health outcomes (Objective 3-3). Precision nutrition approaches will provide the most comprehensive and personalized answers to these objectives when high-quality data from relevant stages are analyzed using multivariate techniques.

## Conclusion

Precision nutrition is an emerging branch of precision medicine. Among the many factors that influence early brain development, nutrition is a relatively modifiable factor. Tremendous opportunities are available to improve neurodevelopmental outcomes by optimizing the very origin of brain development (between conception to infancy), through modifying maternal nutrition (during pre-conception, pregnancy and lactation periods) and infant nutrition. We reviewed the current knowledge in this field, discussed opportunities from data science perspectives, and provided a catalog of major resources. We hope this survey and perspective study helps push nutritional studies for early life to a new height.

## Author Contributions

BL, ET, RV, MS, IL, JL, and MK performed the literature reviews and contributed to the draft. SM, PG, and YO directed the review, acquired funding, and wrote the manuscript with input from all authors. All authors contributed to the article and approved the submitted version.

## Conflict of Interest

JL and MK employees of Abbott Nutrition. The remaining authors declare that the research was conducted in the absence of any commercial or financial relationships that could be construed as a potential conflict of interest.

## Publisher’s Note

All claims expressed in this article are solely those of the authors and do not necessarily represent those of their affiliated organizations, or those of the publisher, the editors and the reviewers. Any product that may be evaluated in this article, or claim that may be made by its manufacturer, is not guaranteed or endorsed by the publisher.
